# Salivary Desmoglein Enzyme-Linked Immunosorbent Assay for Diagnosis of Pemphigus Vulgaris: A Noninvasive Alternative Test to Serum Assessment

**DOI:** 10.1155/2015/698310

**Published:** 2015-01-22

**Authors:** Hossein Mortazavi, Alireza Khatami, Zahra Seyedin, Iman Vasheghani Farahani, Maryam Daneshpazhooh

**Affiliations:** ^1^Autoimmune Bullous Diseases Research Center, Tehran University of Medical Sciences, Tehran 1199663911, Iran; ^2^Department of Dermatology, Razi Hospital, Tehran University of Medical Sciences, Vahdat Islamic Square, Tehran 1199663911, Iran; ^3^Center for Research and Training in Skin Diseases and Leprosy, Tehran University of Medical Sciences, Tehran 1416613675, Iran; ^4^Department of Industrial and Systems Engineering, North Carolina State University, Raleigh, NC 27695, USA

## Abstract

*Background*. Serum desmoglein enzyme-linked immunosorbent assay (ELISA) is used for the diagnosis and monitoring of pemphigus diseases. *Objectives*. To compare the diagnostic accuracy of salivary antidesmoglein (Dsg) 1 and 3 ELISA in the diagnosis of pemphigus vulgaris (PV) patients with that of serum desmogleins ELISA. *Methods*. Eighty-six untreated PV patients and 180 age- and sex-matched PV-free controls were recruited in this case-control study. PV was diagnosed based on clinical, histopathological, and direct immunofluorescence findings. After processing, serum and salivary anti-Dsg 1 and 3 were measured by the ELISA method using Euroimmun kit (Lübeck, Germany). *Results*. Using the cut-off point of 20 relative units (RU)/mL, the serum anti-Dsg 1 and 3 ELISA were positive in 62 (72.1%) and 83 (96.5%) patients, respectively, and the salivary anti-Dsg 1 and 3 ELISA were positive in 31 (36.1%) and 63 (73.3%) patients, respectively. The specificity of salivary anti-Dsg 1 and anti-Dsg 3 were both 98.9%. Optimal cut-off values of 7.7 and 13.4 RU/mL were determined for the salivary anti-Dsg 1 and anti-Dsg 3 ELISA, respectively. *Conclusion*. Salivary anti-Dsg 1 and 3 ELISA with high specificities (98.9%) could be suggested as safe and noninvasive methods for the diagnosis of PV when obtaining a blood sample is difficult.

## 1. Introduction

The usefulness of enzyme-linked immunosorbent assay (ELISA) for the diagnosis of pemphigus vulgaris (PV) has been shown for more than a decade [1]. Today, desmoglein (Dsg) ELISA is routinely used for the diagnosis and monitoring of pemphigus disease [2]. Previously, serum Dsg 1 and 3 ELISA were used for the diagnosis of PV, and a correlation between Dsg ELISA and the severity of the disease was established by our group [3]. Saliva, as a biofluid, could be used for early and noninvasive diagnosis of some diseases [4].

As a preliminary study, our group has already reported the usefulness of saliva anti-Dsg ELISA for the diagnosis of PV using MBL kit (Medical and Biological Laboratories Co. Ltd., Nagoya, Japan) [5].

In the present study, in addition to determination of sensitivities and specificities of different ELISA assessments, we determined several other diagnostic parameters including diagnostic odds ratios, positive likelihood ratios (+LR), negative likelihood ratios (−LR), and Cohen's kappa coefficients to provide a better opportunity for discussing our findings. This approach allowed us to make a more comprehensive judgment considering both clinical and statistical insights.

In this study, we measured salivary and serum anti-Dsg 1 and 3 antibodies in a quite large sample of PV patients and compared them with controls. We aimed to find the diagnostic accuracy of saliva anti-Dsg ELISA in PV patients and then compare it to that of serum anti-Dsg ELISA using Euroimmun kit (Lübeck, Germany).

## 2. Patients and Methods

### 2.1. Participants and Setting

Untreated PV patients, who were referred to or admitted to the dermatology clinics of Razi Hospital which is the main referral skin hospital in Iran located in Tehran, were voluntarily enrolled into the study. The diagnosis of the disease was based on histopathology and direct immunofluorescence findings in favor of PV. Demographic data including gender and age were recorded on a predesigned questionnaire. Clinical characteristics of the disease including the phenotype of disease, namely, cutaneous, mucosal, and mucocutaneous, were recorded as well. Scoring of the disease based on the pemphigus disease area index (PDAI) was also recorded [6].

In order to compare the patients with an appropriate pemphigus-free control group, age- and sex-matched individuals, clinically free of any autoimmune disease based on their medical history and physical examination, and admitted to the Razi Hospital outpatient general dermatology clinic, were also recruited into the study.

### 2.2. Sample Size Calculation

PASS 11 (NCSS, LLC, Kaysville, UT, USA) was used to calculate the needed sample size. Assuming null sensitivity and specificity equal to 0.85 and alternative sensitivity and specificity equal to 0.95, as well as *α* = 0.05, *β* = 0.2, and a case to control ratio equal to 1 : 2, a sample size including 75 cases and 152 controls was needed to test the sensitivities. Since the needed sample size for testing the specificities was smaller (37 cases and 75 controls) we decided to achieve the calculated sample size for testing the sensitivities.

### 2.3. ELISA

One 5-mL blood sample from the antecubital vein and 3 to 5-mL saliva samples were taken from each participant. In order to prevent circadian variations, the serum and saliva samples were obtained from 9:00 to 11:00 AM. The samples were centrifuged for 10 minutes at 3700 g. Thereafter, they were stored at −70°C until performing ELISA.

Anti-Dsg 1 and anti-Dsg 3 serum and salivary ELISA were performed using the Euroimmun (Lübeck, Germany) ELISA kit. Anti-Dsg 1 and anti-Dsg 3 ELISA were performed on the serum samples which were diluted to (1/100) in accordance with the manufacturer instructions. To obtain the optimal dilution for salivary anti-Dsg ELISA, we performed anti-Dsg ELISA on undiluted and two different dilutions, that is, (1/10) and (1/100). We found that the undiluted saliva specimen was optimal. Serum anti-Dsg 1 and anti Dsg-3 ELISA values ≥20 were considered positive results [7, 8].

### 2.4. Statistical Methods

The patients and controls data were collected using a questionnaire, entered into a predesigned dataset using version 16 of the SPSS statistical package (SPSS Inc. Chicago, IL, USA), and were described and analyzed by the aforementioned software and statistical software SigmaPlot 12.5 (Systat Software Inc., San Jose, CA, USA). For statistical description, the continuous data were summarized in tables using mean (SD) for the data with normal distributions, and median (interquartile range [1st–3rd quartile]) for the nonparametric data. Sensitivity, specificity, and positive and negative predictive values of the categorical data were presented as frequencies and relative frequencies (percentages). Pearson correlation was used to assess the score relationship between serum and salivary anti-Dsg 1 and anti-Dsg 3, with disease pemphigus disease activity score. Absolute value of correlation coefficient (*r*) from 0 to 0.25, ≥0.25 and <0.5, ≥0.5 and <0.75, and ≥0.75 was considered little or no relationship, a fair degree of relationship, a good or moderate relationship, and a very good to excellent relationship, respectively. For comparing median for nonparametric data, Mann-Whitney *U* test was used. A *P value* of less than 0.05 was considered as statistically significant.

Receiver operating characteristic (ROC) curves for salivary anti-Dsg 1 and anti-Dsg 3 were plotted and the optimal cut-off value was determined.

## 3. Results

From June 2012 to January 2014, 86 untreated PV patients who were referred to or admitted to the dermatology clinics of Razi Hospital, the main referral skin hospital in Iran, located in Tehran, were enrolled into the study voluntarily. In order to compare the patients with a control group to determine the specificity of serum and saliva tests, 180 age- and sex-matched individuals who were admitted to the Razi Hospital outpatient general dermatology clinic were recruited into the study.

Of 86 patients, 35 (40.7%) were males and 51 (59.3%) were females. The mean (standard deviation; SD) age of the patients was 43.7 (14.8) years. The youngest and oldest patients were 16 and 90 years old, respectively. Phenotypically, 19 patients (22.1%) had mucosal, 5 patients (5.8%) had cutaneous, and the remaining 62 patients (72.1%) had mucocutaneous phenotypes.

### 3.1. Serum Findings

**Figure 1:**
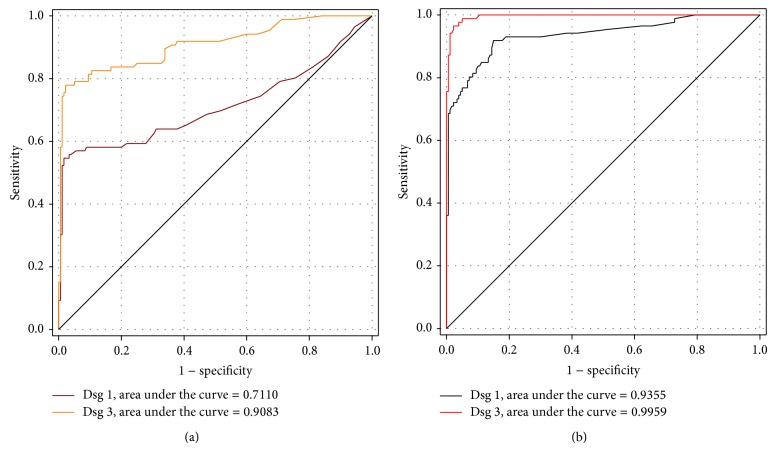
The ROC curve drawn to obtain the optimal cut-off value for salivary (a) and serum (b) Dsg 1 and 3 ELISA.

Using the manufacturer suggested cut-off value of 20 relative units (RU)/mL, the serum anti-Dsg 1 and anti-Dsg 3 ELISA were positive in 62 (72.1%) and 83 (96.5%) of the patients, respectively. The specificities of serum anti-Dsg 1 and anti-Dsg 3 were the same and equaled to 97.8%. We calculated the sensitivity, specificity, +LR, and −LR of serum ELISA with several cut-off points and drew the ROC curve accordingly (Figure 1(b)).

The ROC curve revealed that the manufacturer suggested cut-off value of 20 RU/mL seemed to be the optimal cut-off value for serum anti-Dsg 3 ELISA with a sensitivity of 96.5% and a specificity of 97.8%.

The area under the ROC curve for the serum anti-Dsg 1 and anti-Dsg 3 ELISA were 0.94 (95% CI: 0.90–0.97) and 0.99 (95% CI: 0.99–1.0), respectively. There was a significant difference between these area under curve (AUC) values (*P* < 0.001).

### 3.2. Saliva Findings

With regard to the manufacturer suggested cut-off value for serum anti-Dsg 1 and anti-Dsg 3 ELISA (20 RU/mL), the salivary anti-Dsg 1 and anti-Dsg 3 ELISA were positive in 31 (36.1%) and 63 (73.3%) patients, respectively. The specificity of salivary anti-Dsg 1 and anti-Dsg 3 were both 98.9%.

As the cut-off value of 20 RU/mL was designed for serum anti-Dsg ELISA tests, we examined several cut-off values for salivary anti-Dsg 1 and 3 ELISA using SigmaPlot software (Table 1). The main diagnostic outcomes of the salivary ELISA diagnostic characteristics, including its sensitivity, specificity, +LR, and −LR using different cut-off values, are presented in Table 1.

We drew the ROC curve for salivary anti-Dsg 1 and anti-Dsg 3 data (Figure 1(a)). The area under the ROC curve for the salivary anti-Dsg 1 and anti-Dsg 3 ELISAs were 0.71 (95% CI, 0.63–0.79) and 0.91 (95% CI, 0.87–0.95), respectively. These AUCs were statistically significantly different (*P* < 0.0001).

The frequency of serum and salivary anti-Dsg 1 and anti-Dsg 3 positive ELISA results regarding the PV phenotypes is demonstrated in Table 2.

The median and interquartile range of salivary and serum anti-Dsg 1 and anti-Dsg 3 ELISA values of all participants are demonstrated in Table 3.

### 3.3. Comparison of Dsg Values with Disease Phenotype

According to the data provided in Table 3, comparison of the values of serum and salivary anti-Dsg 1 and anti-Dsg 3 ELISA in PV patients regarding the phenotype of their disease was as follows.

1. *Mucosal versus Cutaneous Phenotypes.* Patients with the cutaneous PV phenotype had larger values of serum anti-Dsg 1 than those with mucosal phenotype (*P* = 0.009). In addition, those with mucosal phenotype had larger values of salivary anti-Dsg 3 than the patients with cutaneous phenotype (*P* = 0.043).
2. *Mucosal versus Mucocutaneous Phenotypes*. PV patients with mucocutaneous phenotype had larger values of anti-Dsg 1 and anti Dsg 3 in both saliva and serum in comparison with those with mucosal phenotype (serum anti-Dsg 3 and saliva anti-Dsg 1, *P* = 0.001 and serum anti-Dsg 1 and saliva anti-Dsg 3, *P* < 0.001).
3. *Cutaneous versus Mucocutaneous Phenotype.* PV patients with the mucocutaneous phenotype had larger values of salivary anti-Dsg 3 than those with cutaneous phenotype (*P* = 0.002).

### 3.4. Correlation of Anti-Dsg Values with Age and Gender

There was a negative association between serum anti-Dsg 1 and the age of patients (*r* = −0.287, *P* = 0.007). Other correlation tests revealed nonsignificant associations.

### 3.5. Correlation of Serum and Salivary Dsg Values

The correlation of serum and salivary anti-Dsg 1 and anti-Dsg 3 values in all PV patients and those with the mucocutaneous phenotype were investigated. The statistically significant correlations are demonstrated in Table 4.

### 3.6. Correlation of Serum and Salivary Dsg Values with Pemphigus Score (PDAI)

The serum and salivary anti-Dsg 1 and anti-Dsg-3 values were significantly correlated with the PV total score (the sum of total skin score and mucosal score; *P* < 0.05 for all correlations). Similarly, the serum and salivary anti-Dsg 1 and anti-Dsg 3 values were significantly correlated with the total skin score (the sum of skin score and scalp score; all *P* < 0.05 for all correlations).

In addition, the salivary anti-Dsg 1 and anti-Dsg 3 values were significantly correlated with the mucosal score (*r* = 0.257 and *r* = 0.277 with *P* = 0.017 and *P* = 0.010, resp.).

In cutaneous phenotype, the serum Dsg 1 values were significantly correlated with the total score (*r* = 0.973, *P* = 0.005).

In the mucocutaneous phenotype, the salivary Dsg 1 and 3 values were significantly correlated with the total score (*r* = 0.346 and *r* = 0.347, respectively, and both *P* = 0.006).

## 4. Discussion

A recent systematic review and meta-analysis has shown that anti-Dsg ELISA is a valuable laboratory diagnostic method for the initial diagnosis of autoimmune bullous diseases including PV and could be used in daily practice [9].

Saliva could be used as a window for observation of the state of a human being's health and the detection of diseases [10]. In view of new technologies, namely, nanotechnology, proteomics, and genomics, diagnostic methods using saliva have been proven to be highly sensitive [11]. Serum components such as antibodies, cytokines, and hormones could be transferred to saliva through capillary walls in the salivary glands [12]. Secretory IgA and IgG are the two principal antibodies in saliva [13]. Moreover, a significant association of saliva IgG with its serum concentration has been shown in inflammatory conditions [13]. Applying the ELISA method, IgG autoantibodies to Dsg 1 and Dsg 3 have already been determined [1, 14]. An animal study showed that the concentration of IgG in saliva is much lower than that of serum [15]. Therefore, in order to detect IgG autoantibodies to Dsg 1 and Dsg 3, we used undiluted saliva, while according to the manufacturer's instruction we used sera diluted to (1/100) for the ELISA tests.

In the present study, we intended to investigate the diagnostic accuracy of serum and saliva anti-Dsg ELISA as specific tests for the diagnosis and monitoring of PV [9, 16]. We also aimed to understand whether it could be a substitute for serum ELISA in certain circumstances.

### 4.1. Serum Studies

Using the suggested manufacturer cut-off value of 20 RU/mL for serum anti-Dsg 1 and anti-Dsg 3 ELISA, the sensitivity of serum Dsg ELISA in untreated PV patients in our study was 72.1% for anti-Dsg 1 and 96.5% for anti-Dsg 3, respectively. Harman et al. reported a sensitivity of serum anti-Dsg 3 ELISA in untreated PV patients equal to 100% (34/34) [17]. In our previous studies, we reported sensitivities for serum anti Dsg 3 ELISA (using MBL kits) equaled to 88.9% (24/27), 94% (47/50), and 94.5% (69/73) [3, 5, 18]. The sensitivities of serum anti-Dsg 1 ELISA in untreated PV patients in our previous studies were 70.4% (19/27), 72% (36/50), and 76.7% (56/73) [3, 5, 18]. Tampoia et al. also showed that serum anti-Dsg antibodies are highly sensitive and specific for the diagnosis of PV [9].

According to McKibbon, a test with an area under the ROC curve greater than 0.80 is considered to be a “good” test [19]. Our study confirmed the serum anti-Dsg 1 and anti-Dsg 3 ELISA with area under the ROC curve of 0.94 and 0.99, respectively, to be good tests.

According to Table 5, serum anti-Dsg 3 ELISA with a high sensitivity and specificity (96.5% and 97.8%) and with other appropriate values is a good diagnostic test for PV. Serum anti-Dsg 1 ELISA also yielded good and comparable results, but with a lower sensitivity. Both serum anti-Dsg 1 and anti-Dsg 3 ELISA had a high diagnostic odds ratio (113.7 and 1217.3) as well as a high area under the ROC curve (0.94 and 0.99) making them good diagnostic tools for differentiating the diseased from nondiseased conditions [19, 20]. Several other studies also confirmed the usefulness of these tests for the diagnosis and monitoring of pemphigus [1, 18, 21–24].

### 4.2. Saliva Studies

The sensitivity of salivary Dsg 1 and 3 ELISA in the present study with the cut-off value of 20 RU/mL was 36.1% and 73.3%, respectively. Using another study as a framework, we drew the ROC curve of salivary anti-Dsg 1 and anti-Dsg 3 ELISA (Figure 1(a)) to obtain the best cut-off value [23]. According to Table 1, the cut-off values of 8.9, 13.4, and 20 RU/mL for salivary anti-Dsg 3 ELISA yielded the sensitivity and specificity of (80.2% and 90.6%), (77.9% and 97.8%), and (73.3% and 98.9%), respectively.

Previously, the value of serum anti-Dsg 3 has been shown for the diagnosis and management of PV [25]. We used different measurements (AUC, Cohen's kappa and +LR) to investigate whether the salivary anti-Dsg 3 ELISA could be an appropriate alternative for the diagnosis of PV as follows.

1. Salivary anti-Dsg 3 ELISA with area under the ROC curve of 0.91 could be considered a good test for the diagnosis of PV [19].
2. Cohen's kappa coefficient is a statistical measure to calculate the level of agreement between the combination of histopathological and DIF examinations as the gold standard test for the diagnosis of PV and the salivary anti-Dsg 3 ELISA as an index test (test under study) [26]. With all cut-off values presented in Table 1 for salivary Dsg 3 ELISA, a “perfect agreement” existed between the combination of histopathology and DIF examinations, and salivary anti-Dsg 3 ELISA (Cohen's kappa > 0.8) [26].
3. According to some authors [16, 19], a test with a +LR greater than 5 is clinically useful for diagnosing the condition of interest, in the present study, diagnosis of PV. For all cut-off values presented in Table 1, salivary anti-Dsg 3 ELISA have high +LR.

According to Khatami and Gorouhi, in general, a “good” test has a sensitivity and specificity higher than 80% [16]. In our study, the cut-off value of 8.9 RU/mL could be considered the optimal cut-off value for salivary anti-Dsg 3 ELISA, since it had a sensitivity and specificity higher than 80%. According to Glas et al., a test with the high diagnostic odds ratio (DOR) can discriminate more precisely between the patients with PV and the control group [20]. In this regard, salivary anti-Dsg 3 ELISA at the cut-off point value of 13.4 RU/mL is much more specific in comparison with the cut-off point value of 8.9 RU/mL and resulted in a higher DOR which actually makes the former cut-off point of 13.4 RU/mL a more suitable one. As previously discussed, this value (13.4 RU/mL) is a clinically useful and suitable cut-off point which has a +LR > 5 and a Cohen's kappa > 0.8.

In Table 1, three cut-off values for the salivary anti-Dsg 1 ELISA test are presented, namely, 5.4, 7.7, and 20 RU/mL. To choose the most appropriate cut-off point we considered the following. First, the cut-off values of 7.7 and 20 RU/mL have the same specificity of 98.9% while the former has a higher sensitivity. Thus, a cut-off point of 7.7 RU/mL could be more appropriate than 20 RU/mL. Second, several major outcomes of the salivary anti-Dsg 1 ELISA test, that is, sensitivity, specificity, positive and negative predictive values, and test accuracy with two cut-off values of 5.4 and 7.7 RU/mL, were quite similar. Considering DOR of the test, the cut-off value of 7.7 RU/mL with the DOR of 97.7 would be the most appropriate point as it can discriminate between the patients with PV and the control group more precisely [20]. Our data revealed that there is perfect agreement between pathology and DIF, and salivary anti-Dsg 1 ELISA at the cut-off point of 7.7 RU/mL (Cohen's kappa = 0.83) [26].

According to the above discussion, we concluded that cut-off value of 13.4 RU/mL and 7.7 RU/mL are the optimal cut-off points for the diagnosis of PV using salivary anti-Dsg 3 and anti-Dsg 1 ELISA, respectively. Since the area under the ROC curve for salivary anti-Dsg 1 ELISA was equal to 0.71 and was significantly smaller than that of salivary anti-Dsg 3 ELISA (*P* < 0.0001), we can infer that salivary anti-Dsg 3 ELISA is a better test than salivary anti-Dsg 1 ELISA for the diagnosis of PV for diagnostic purposes.

Interestingly, salivary anti-Dsg 3 ELISA with a cut-off value of 20 RU/mL revealed very similar results to serum anti-Dsg 1 ELISA (Table 5).

Being a noninvasive test, salivary anti-Dsg 3 ELISA might be a suitable substitute for the serum anti-Dsg ELISA tests in the diagnosis of PV.

### 4.3. Correlation of Dsg Values with Pemphigus Score (PDAI)

In the present study, the serum anti-Dsg 1 was significantly correlated with the total skin score. Our previous studies showed that serum anti-Dsg 1 was correlated with severity of skin involvement [3, 5, 18]. In addition, the present study showed that serum anti-Dsg 1 and anti-Dsg 3 values were significantly correlated with total score of the disease.

Undiluted salivary anti-Dsg 3 ELISA assessment revealed comparable results to those of serum Dsg 1 ELISA (with (1/100) dilution). Since the salivary anti-Dsg 3 ELISA is highly specific for the diagnosis of PV and has an acceptable accuracy, it might be suggested for monitoring PV patients.

In conclusion, the salivary anti-Dsg 1, and in particular anti-Dsg 3 ELISA, could be used as safe and noninvasive methods for the diagnosis of PV, when obtaining a blood sample is difficult (in certain circumstances, e.g., pediatric and senile patients). Cut-off values of 7.7 and 13.4 RU/mL are the most appropriate ones for the salivary anti-Dsg 1 and anti-Dsg 3 ELISA, respectively.

## Figures and Tables

**Table 1:** Salivary anti-Dsg 1 and anti-Dsg 3 ELISA tests main diagnostic outcomes using three different cut-off values.

	Salivary anti-Dsg 1 ELISA			Salivary anti-Dsg 3 ELISA		
Cut-off (RU/mL)	5.4	7.7	20	8.9	13.4	20
True positive	48	45	31	69	67	63
True negative	174	178	178	163	176	178
False positive	6	2	2	17	4	2
False negative	38	41	55	17	19	23
Sensitivity	55.8%	52.3%	36.1%	80.2%	77.9%	73.3%
Specificity	96.7%	98.9%	98.9%	90.6%	97.8%	98.9%
Positive predictive values	0.89	0.96	0.94	0.80	0.94	0.97
Negative predictive values	0.82	0.81	0.76	0.91	0.90	0.89
Test accuracy	0.83	0.84	0.79	0.87	0.91	0.91
Positive likelihood ratio (+LR)	16.7	47.1	32.4	8.5	35.1	65.9
Negative likelihood ratio (−LR)	0.5	0.5	0.6	0.2	0.2	0.3
Diagnostic odds ratio	36.6	97.7	50.2	38.9	155.2	243.8
Cohen's kappa coefficient	0.82	0.83	0.78	0.86	0.91	0.90

**Table 2:** Frequency of positive results of salivary and serum anti-Dsg 1 and anti-Dsg 3 ELISA in PV patients according to their disease phenotype.

Disease phenotype	*N* (%)	Saliva ELISA		Serum ELISA	
		Anti-Dsg 1 (%)	Anti-Dsg 3 (%)	Anti-Dsg 1 (%)	Anti-Dsg 3 (%)
Mucosal	19 (22.1%)	2 (10.5%)	12 (63.2%)	6 (31.6%)	17 (89.5%)
Cutaneous	5 (5.8%)	1 (20.0%)	1 (20.0%)	4 (80.0%)	4 (80.0%)
Mucocutaneous	62 (72.1%)	28 (45.2%)	50 (80.6%)	52 (83.9%)	62 (100.0%)

Total	86 (100.0%)	31 (36.0%)	63 (73.3%)	62 (72.1%)	83 (96.5%)

**Table 3:** The values of salivary and serum anti-Dsg 1 and anti-3 ELISA in PV patients regarding their phenotype and controls.

Disease phenotype	Salivary ELISA		Serum ELISA	
	Anti-Dsg1^*^	Anti-Dsg3^*^	Anti-Dsg1^*^	Anti-Dsg3^*^
	Median (IQR^**^)	Median (IQR)	Median (IQR)	Median (IQR)
Mucosal	1.80 (1.3–6.9)	21 (8.3–92)	3.2 (2.3–28)	163 (40–322)
Cutaneous	2.40 (1.75–80.4)	3.8 (1.6–14.8)	186 (94.15–376.5)	165 (32.15–358.5)
Mucocutaneous	17.35 (2.15–114.25)	182 (35.75–255)	136 (58.5–241.75)	310 (244.5–399.25)
Overall	8.4 (1.7–75.25)	102.5 (16.925–215.25)	90 (12.225–213.25)	297 (200.25–382)
Controls	2 (1.6–3.1)	2.1 (1.4–4.8)	1.5 (1.1–1.9)	1.2 (1.1–1.6)

^*^The unit for measurement is RU/mL.

^**^Interquartile range.

**Table 4:** Pearson's correlation of serum and salivary Dsgs values in all 86 PV patients and 62 PV patients with mucocutaneous phenotype.

	Values^*^		Correlation coefficient (*r*)	*P *
In all patients (*N* = 86)	Serum anti-Dsg-1	Serum anti-Dsg-3	0.332	0.002
	Saliva anti-Dsg-1	Saliva anti-Dsg-3	0.629	<0.001
	Serum anti-Dsg-1	Saliva anti-Dsg-1	0.337	0.001
	Serum anti-Dsg-3	Saliva anti-Dsg-3	0.435	<0.001

In patients with mucocutaneous phenotype (*N* = 62)	Serum anti-Dsg-1	Serum anti-Dsg-3	0.271	0.033
	Saliva anti-Dsg-1	Saliva anti Dsg-3	0.659	<0.001
	Serum anti-Dsg-1	Saliva anti Dsg-1	0.319	0.012
	Serum anti-Dsg-3	Saliva anti Dsg-3	0.353	0.005

^*^Values (RU/mL) have been provided in Table 2.

**Table 5:** Serum anti-Dsg 1, serum anti-Dsg3, and salivary antidesmoglein 3 ELISA main diagnostic outcomes using the manufacturer's suggested cut-off value of 20 RU/mL.

	Serum anti-Dsg 1 ELISA	Serum anti-Dsg 3 ELISA	Salivary anti-Dsg 3 ELISA
True positive	62	83	63
True negative	176	176	178
False positive	4	4	2
False negative	24	3	23
Sensitivity	72.09%	96.51%	73.26%
Specificity	97.78%	97.78%	98.89%
Positive predictive values	0.94	0.95	0.97
Negative predictive values	0.88	0.98	0.89
Test accuracy	0.89	0.97	0.91
Positive likelihood ratio	32.44	43.33	65.93
Negative likelihood ratio	0.29	0.04	0.27
Diagnostic odds ratio	113.67	1217.33	243.78
Cohen's kappa coefficient	0.89	0.97	0.90
